# iTRAQ-based comparative proteomic analysis of cells infected with *Eimeria tenella* sporozoites

**DOI:** 10.1051/parasite/2019009

**Published:** 2019-02-20

**Authors:** Zongping Zhao, Qiping Zhao, Shunhai Zhu, Bing Huang, Ling Lv, Ting Chen, Ming Yan, Hongyu Han, Hui Dong

**Affiliations:** Key Laboratory of Animal Parasitology of Ministry of Agriculture, Shanghai Veterinary Research Institute Shanghai 200241 PR China

**Keywords:** iTRAQ, *Eimeria tenella*, Sporozoites, BHK-21 cells, Differentially expressed proteins

## Abstract

*Eimeria tenella* is an obligate intracellular parasite that actively invades cecal epithelial cells of chickens. When *E. tenella* infects a host cell, the host produces a corresponding change to deal with damage caused by this infection. To date, our knowledge on the mechanism of how the host cell responds to *E. tenella* infection is highly limited at both the molecular and cellular levels. In this study, isobaric tags for relative and absolute quantitation (iTRAQ) coupled with LC-MS/MS was used to screen the differentially expressed proteins (DEPs) in BHK-21 cells infected with *E. tenella* sporozoites for 24 h post infection. In total, 6139 non-redundant distinct proteins were identified and 195 of these were found to have a fold change ratio ≥1.3 or ≤0.7 and *p* < 0.05, including 151 up-regulated proteins and 44 down-regulated proteins. The reliability of the proteomic data was further validated with qPCR and western blot. Gene Ontology enrichment indicated that the up-regulated DEPs were mainly involved in binding and catalytic activity, whereas the down-regulated DEPs were catalytic activity and molecular function regulators. Furthermore, KEGG pathway analysis showed that the DEPs participated in the PI3K-Akt, chemokine, Ras, Wnt, and p53 signaling pathways and so on, and the up-regulated and down-regulated DEPs mainly related to the ribosome and mRNA surveillance pathway, respectively. The data in this study provide an important basis to further analyze *E. tenella* host cell interactions.

## Abstract (fr)

*Eimeria tenella* est un parasite intracellulaire obligatoire qui envahit activement les cellules épithéliales cæcales des poulets. Quand *E. tenella* infecte une cellule, l’hôte produit un changement correspondant pour traiter les dommages causés par cette infection. À ce jour, nos connaissances sur le mécanisme de réponse de la cellule hôte à l’infection à *E. tenella* sont très limitées, tant au niveau moléculaire que cellulaire. Dans cette étude, des marqueurs isobares pour la quantification relative et absolue (iTRAQ) couplés à la LC-MS / MS ont été utilisés pour cribler les protéines exprimées de manière différentielle (PED) dans des cellules BHK-21 infectées par des sporozoïtes de *E. tenella*, 24 h après l’infection. Au total, 6139 protéines distinctes non redondantes ont été identifiées, et 195 d’entre elles présentaient un taux de variation de ≥ 1,3 ou ≤ 0,7 et un *p* < 0,05, dont 151 protéines régulées positivement et 44 protéines régulées négativement. La fiabilité des données protéomiques a ensuite été validée avec qPCR et Western Blot. L’enrichissement par l’ontologie des gènes a indiqué que les PED régulés positivement étaient principalement impliquées dans la liaison et l’activité catalytique, alors que les PED régulées négativement étaient celles de l’activité catalytique et des régulateurs de la fonction moléculaire. De plus, l’analyse de la voie de KEGG a montré que les PED participaient aux voies de signalisation de PI3K-Akt, de la chémokine, de Ras, de Wnt, de p53, et autres, et que les PED régulées positivement et négativement étaient liées respectivement aux voies de surveillance du ribosome et de l’ARNm, respectivement. Les données de cette étude fournissent une base importante pour une analyse plus poussée des interactions entre les cellules hôtes d’*E. tenella*.

## Introduction

Coccidiosis of the chicken is caused by the genus *Eimeria* and is one of the most common and serious diseases on farms worldwide [14]. Coccidiosis causes serious economic losses in poultry farming [25]. Currently, the prevention and control of chicken coccidiosis mainly relies on anti-coccidial drugs, but drug-resistance has become an unavoidable problem. Therefore, there is an urgent need to find new measures to control chicken coccidiosis [24, 30].

*Eimeria* has a complex life cycle and its development and reproduction depend on the intestinal epithelium of chickens. The invasion of host intestinal epithelium by *Eimeria* species is a complex, multistep process. It starts with the adhesion of the parasite to the host cell. Then by quick invasion, it takes the shape of an intracellular, parasitophorous vacuole that surrounds the newly invading parasite, ensuring its survival within the host [27]. In order to perpetuate the infection, *Eimeria* first egress from infected cells and then reinvade uninfected cells. Faced with these circumstances, host cells have formed their own regulatory mechanisms to cope with these changes and invasion. Specifically, the expression of IDO1 (indoleamine 2,3-dioxygenase1) in the host epithelial cells increases after being infected with *E. falciformis* and this level of expression remains during the entire infection with the parasite. This response plays a vital role in maintaining the parasite at optimal development [22]. Additionally, *F*-actin has been shown to accumulate in human HCT-8 ileocecal adenocarcinoma cells after being infected with *E. tenella*, and plays a positive role in parasitic invasion [32]. Furthermore, chicken Toll-like receptor 4 (ChTLR4) and ChTLR15 have been shown to be up-regulated and involved in the recognition of *E. tenella* after infection [36]. Taken together, these results show that *Eimeria* have the ability to reprogram host cell response for their survival and reproduction. However, the extent and relevance of *Eimeria*-mediated host responses remains poorly studied.

Isobaric tags for relative and absolute quantitation (iTRAQ) in combination with liquid chromatography tandem mass spectrometry (LC-MS/MS) analysis have become an important quantitative proteomic method with certain advantages over traditional proteomic techniques. These advantages include higher throughput, increased sensitivity, and greater accuracy. This technique has been used successfully to explore pathogen–host interactions for both viruses [7] and bacteria [37]. In our study, iTRAQ was used to identify the differentially expressed proteins (DEPs) in Baby Hamster Kidney fibroblast cells (BHK-21) infected with *E. tenella* sporozoites.

## Materials and methods

### Ethics statement

All animal procedures were ratified by the Animal Ethics Committee of the Shanghai Veterinary Institute, Chinese Academy of Agricultural Science. Experiments were carried out according to animal ethics guidelines and approved protocols.

### Parasite and cell culture of BHK-21

The Shanghai strain of *E. tenella* was isolated from a sample collected on a chicken farm in Shanghai, China, and has been retained in our laboratory since 1993. *E. tenella* was propagated by means of coccidia-free 2-week-old chickens, as described previously [29]. Sporozoites were prepared from cleaned sporulated oocysts by *in vitro* excystation and purified by chromatography over columns packed with nylon wool and DE-52 cellulose [35]. BHK-21 was cultured in Dulbecco’s modified Eagle’s medium (DMEM) (Life Technology, Carlsbad, CA, USA) supplemented with 10% fetal bovine serum (FBS) at 37 °C and 5% CO_2_.

### Sample collection

Monolayers of BHK-21 cells (1 × 10^6^ cell per flask) were seeded into T25 culture flasks and cultured to 80% conﬂuence. Freshly excysted sporozoites were treated with penicillin/streptomycin, then centrifuged at 1000 ×*g* for 5 min, washed twice with DMEM, and quantified using a hemocytometer. The pretreated sporozoites were added to adherent cells at a ratio of two sporozoites per cell and cultured at 41 °C in 5% CO_2_. At the same time, the mock-infected cell samples were set as a control group. The *E. tenella*- and mock-infected cells were collected at 24 hpi. Each group was treated with three independent biological replicates.

### Protein extraction

The *E. tenella*- and mock-infected cell samples were washed with chilled phosphate-buffered saline (PBS) three times, collected with cell scrapers, and homogenized in lysis buffer (4% SDS, 1 mM DTT, 150 mM *Tris*-HCl pH 8.0, protease inhibitor), then incubated for 3 min in boiling water and sonicated on ice twice, and clarified by centrifugation at 16,000 ×*g* at 25 °C for 10 min. Protein content was determined using the BCA protein assay reagent (Beyotime) and stored at −80 °C until use.

### Protein digestion and iTRAQ labeling

Protein digestion was conducted by the filter-aided sample preparation (FASP) procedure described previously [31]. The acquired peptide mixture was labeled using the 8-plex iTRAQ reagent, according to the manufacturer’s instructions (AB SCIEX, Foster City, CA, USA). In short, for each sample, 200 μg of protein was added to 30 μL SDT buffer (4% SDS, 100 mM DTT, 150 mM *Tris*-HCl pH 8.0). DTT and other low-molecular-weight components were removed with UA buffer (8 M Urea, 150 mM *Tris*-HCl pH 8.0) by repeated ultrafiltration (Pall units, 10 kDa). Then, 100 μL iodoacetamide (0.05 M in UA) was added to the sample to block reduced cysteine residues and incubated for 20 min in darkness. The filters were washed with 100 μL of UA buffer three times, and then with 100 μL DS buffer (50 mM triethylammonium bicarbonate at pH 8.5) twice. Finally, the protein suspensions were digested with 2 μg trypsin (Promega, Madison, WI, USA) in 40 μL DS buffer overnight at 37 °C and the resulting peptides were collected as a filtrate. The peptide content was estimated with UV light spectral density at 280 nm using an extinction coefficient of 1.1 for a 0.1% (g/L) solution that was calculated on the basis of the frequency of tryptophan and tyrosine in vertebrate proteins.

For labeling, each iTRAQ reagent was dissolved in 70 μL of ethanol and added to the respective peptide mixture. The mock-infected groups were labeled with iTRAQ tag 113, 114 and 115; and the *E. tenella*-infected groups were labeled with iTRAQ tag 116, 117 and 118. The labeled samples were incubated at room temperature for 2 h and then terminated, then mixed and dried with a rotary vacuum concentrator.

### Peptide fractionation with strong cation exchange (SCX) chromatography

iTRAQ labeled peptides were fractionated with SCX chromatography using the AKTA Purifier system (GE Healthcare, Chalfont Saint Giles, UK). The dried peptide mixture was reconstituted and acidified with 2 mL of buffer A (10 mM KH_2_PO_4_ in 25% ACN, pH 2.7) and loaded onto a PolySULFOETHYL 4.6 × 100 mm column (5 μm, 200 Å, PolyLC Inc., Maryland, MD, USA). The flow rate was kept at 1 mL/min, the gradient program started with 0–10% buffer B (500 mM KCl, 10 mM KH_2_PO_4_ in 25% ACN, pH 2.7) for 2 min, followed by 10–20% buffer B for 25 min, 20–45% buffer B for 5 min, and 50–100% buffer B for 5 min. Eluting peptides were monitored at 214 nm and fractions were collected over one-minute intervals. The collected fractions (about 30 fractions) were finally combined into 10 pools and a C18 clean-up (Empore™ SPE Cartridges C18 standard density, bed I.D. 7 mm, volume 3 mL, Sigma, Tokyo, Japan) was performed according to the manufacturer’s instructions, then concentrated by vacuum centrifugation and reconstituted in 40 μL of 0.1% (v/v) trifluoroacetic acid. All samples were stored at −80 °C until further use.

### Liquid chromatography (LC)-electrospray ionization tandem MS (MS/MS) analysis with Q Exactive

Experiments were performed on a Q Exactive mass spectrometer that was coupled to Easy nLC (Thermo Fisher Scientific, Waltham, MA, USA). Each fraction (10 μL) was injected for nanoLC-MS/MS analysis. The peptide mixture (5 μg) was loaded onto a C18-reversed phase column (15 cm long, 75 μm inner diameter) packed in-house with RP-C18 5 μm resin in buffer A (0.1% formic acid) and separated with a linear gradient of buffer B (80% acetonitrile and 0.1% formic acid) at a flow rate of 250 nL/min controlled by IntelliFlow technology over 60 min. MS data were acquired using a data dependent top 10 method, dynamically choosing the most abundant precursor ions from the survey scan (300–1800 m/z) for higher-energy C-trap dissociation (HCD) fragmentation. Determination of the target value was based on predictive automatic gain control. The dynamic exclusion duration was 60 s. Survey scans were acquired at a resolution of 70,000 at 200 m/z and resolution for HCD spectra was set to 17,500 at 200 m/z. Normalized collision energy was 30 eV, which specifies the minimum percentage of the target value likely to be reached at maximum fill time, and was defined as 0.1%. The instrument was run with peptide recognition mode enabled. All the experiments included three biological replicates, and each contained two technical replicates.

### Sequence database searching and data analysis

MS/MS spectra were searched using MASCOT engine (Matrix Science, London, UK; version 2.2) embedded into Proteome Discoverer 1.4 (Thermo Electron, San Jose, CA, USA) against the UniProt Cricetidae database (74,901 sequences, download on April 10, 2017) and the decoy database. For protein identification, the following options were used. Peptide mass tolerance = 20 ppm, MS/MS tolerance = 0.1 Da, Enzyme = Trypsin, Missed cleavage = 2, Fixed modification: Carbamidomethyl (C), iTRAQ8plex (K), iTRAQ8plex (N-term), Variable modification：Oxidation (M), FDR ≤ 0.01.

### Bioinformatics

The Gene Ontology (GO) program Blast2GO (https://www.blast2go.com/) was used to annotate differential expression proteins to create histograms of GO annotation, including cell component, biological process, and molecular function. For pathway analysis, the differentially proteins were mapped to the terms in the KEGG (Kyoto Encyclopedia of Genes and Genomes) database by using the KAAS program (http://www.genome.jp/kaas-bin/kaas_main).

### qPCR analysis

Quantitative real-time PCR (qPCR) was employed to compare gene expression in *E. tenella*-infected and mock-infected cells. Total RNA was extracted with the TRIzol reagent (Invitrogen) and treated with DNase (Invitrogen). cDNA was synthesized from 2 μg of total RNA by SuperScript II reverse transcriptase (Invitrogen) using random primers. Real-time PCR was performed on a Rotor-Gene 3000 (Corbett Robotics, San Francisco, CA, USA) using the SYBR1 green I dye method and speciﬁc primer sets (Table 1). The amplification reactions were performed with the following conditions: 30 s at 95 °C, 40 cycles of 95 °C for 5 s, 60 °C for 30 s and 72 °C for 30 s. Relative transcript levels were calculated using the 2^−∆∆CT^ method [23]. The relative expression values of the targeted gene were normalized to the expression value of the BHK-21 glyceraldehyde-3-phosphate dehydrogenase (GAPDH) gene. All the experiment included three biological replicates and each contained three technical replicates.

**Table 1:** Sequence of gene-specific primers for qPCR assays.

Gene name	Primer sequence (5′ – 3′)
Atp51	UP – AGCCTGACCTTGGAACTGGG, LP – CAGGGATTTCAGCAGGGGTT
Mapk9	UP – CCCTATGTGGTGACTCGCTAT, LP – ACATAATTCCTCACGGTTGG
Ptp4a1	UP – GGCTGCTGTATTGCTGTCCA, LP – CATCAGGCACCCCAGTTTTA
Bnip2	UP –GCCAACCTGGCTCATTAGAA, LP – CAAATCATCCGACCACACAG
Pdss2	UP – GCAGATTGGAGAGGCTCAAG, LP – CTCCAGTGCCTTGTTTCCAT
Ppp2r4	UP – TTGTGGATGAGAAGGCAGTG, LP – GGTCCACAGCTGGTTGGAGT
Gdil	UP – GGACAGGTCTTACCGAATGC, LP – ATGCCCATCAGATTAGAAGC
Sec61a1	UP – TCAGCCCCACCACTGTCAAC, LP – TCGGAAGCCCTGGAAATAGA
Tm7sf2	UP – CTCGCCTTGGTTCCTTTGAC, LP – CAGAGGGCATCACCCACATA
Rps8	UP – GGGCACTCGCTACATGTTCT, LP – GCCCATTCCCTTGATGTCTA
Mrp120	UP – GGAGGTGCTGAAACATGCTC, LP – GTTCCGCTTCTTCAGTCTGC
Tfrc	UP – CCAGTGTTGGAACAGGTCTT, LP – CTCCGAAGTCTCCTGCACTC
GAPDH	UP – TGTGGAAGGACTCATGACCA, LP – GGATGCAGGGATGATGTTCT

### Western blotting

We analyzed the Lamin-B receptor (LBR) and Metastasis-associated protein MTA3 (MTA3) by western blotting because clear variation in their protein expression levels could be detected. Cell lysates for *E. tenella*-infected and mock-infected cultures were harvested at 24 hpi and protein concentrations were determined using the QuantiPro™ BCA Assay Kit (Sigma), according to the manufacturer^’^s instructions. Equivalent quantities of protein were subjected to SDS-PAGE and then transferred to polyvinylidene difluoride transfer membranes (Millipore, Boston, MA, USA). The membranes were blocked in Phosphate Buffer Solution with Tween 20 (PBST) containing 1% BSA for 2 h at 37 °C, after which they were incubated overnight at 4 °C with primary antibodies to LBR, MTA3 and β-actin (CUSABIO, Wuhan, China). The membranes were washed five times with PBST and then incubated with HRP-conjugated secondary antibodies (CUSABIO) at 37 °C for 1 h. The membranes were washed ﬁve times and proteins were then detected using Clarity ECL reagents (Amersham Biosciences, Waukesha, WI, USA).

## Results

### Protein proﬁling of BHK-21 cells

**Figure 1:**
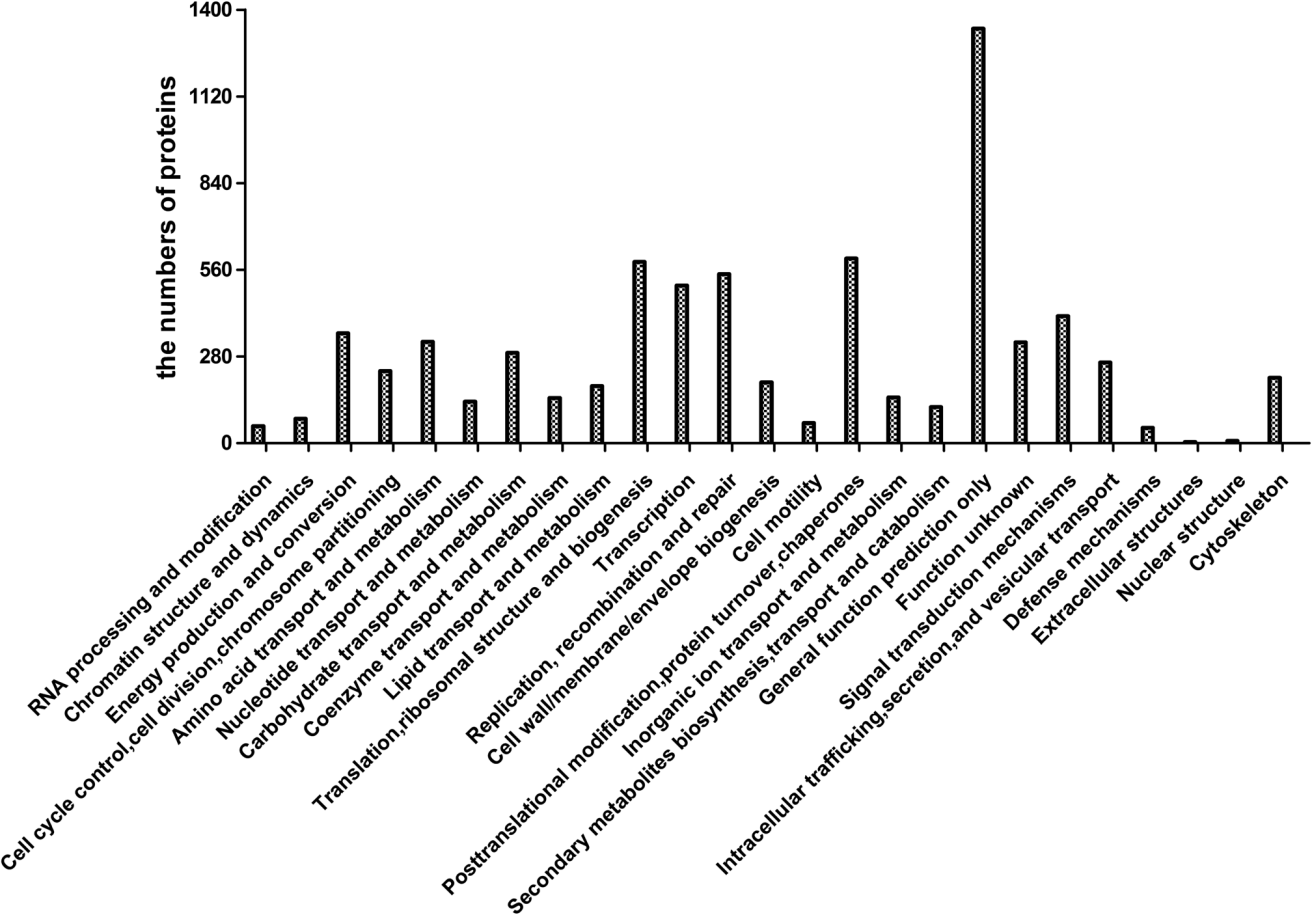
Gene ontology annotations of the proteome of BHK cells infected with *Eimeria tenella.*

After processing the MS/MS spectra using Mascot software, 30,984 unique peptides were mapped to 6139 proteins from BHK-21 cells, which were assigned to 25 different GO categories. The most abundantly populated GO category with 1339 proteins was General Function (Fig. 1). Other frequently assigned categories included post-translational modiﬁcation, protein turnover, chaperones (596 proteins), translation, ribosome structure, and biogenesis (585 proteins), amino acid transport and metabolism (328 proteins), and energy production and conversion (356 proteins).

### Protein responses to *E. tenella* infection in BHK-21 cells

Based on a cut-off of a fold change ≥1.3 or ≤0.7 and a *p* < 0.05 [20, 38], 195 proteins were found to be signiﬁcantly changed in BHK-21 cells in response to *E. tenella* infection (Supplementary Table S1). This included 151 up-regulated proteins and 44 down-regulated proteins. The top five up-regulated DEPs were identified as serine/threonine-protein phosphatase 2A activator, BCL2/adenovirus E1B 19 kDa protein-interacting protein 2, Plexin-D1, decaprenyl-diphosphate synthase subunit 2-like protein, and an uncharacterized protein. The top five down-regulated DEPs were found to be collagen alpha-1(XXVII) chain-like protein, BAG family molecular chaperone regulator 3, telomerase protein component 1, and two uncharacterized proteins. Taken together, these results indicate that *E. tenella* invasion induced a distinct proteomic proﬁle in BHK-21 cells, and in turn host cells sharply altered the related proteins in response to *E. tenella* infection.

**Figure 2:**
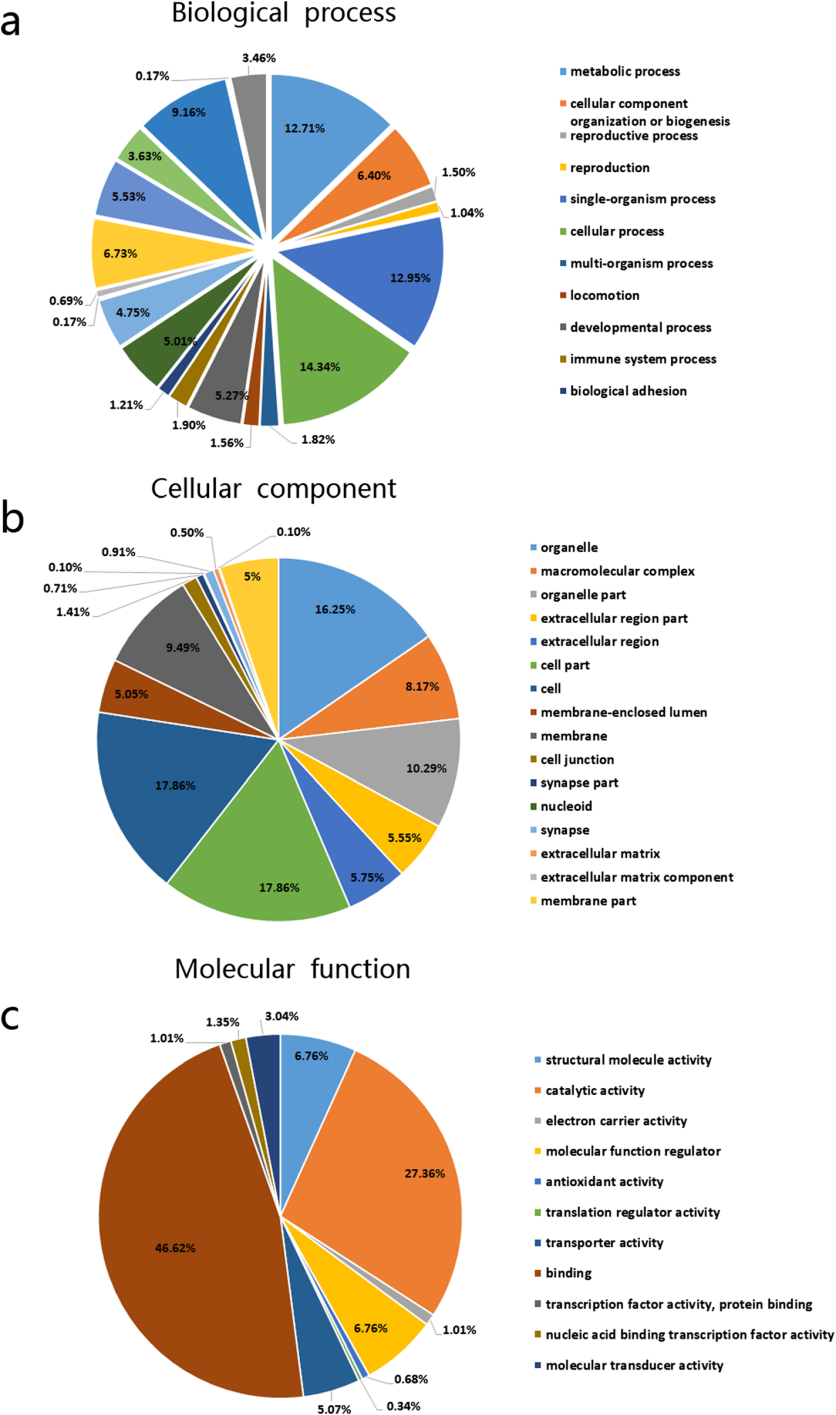
Gene ontology analysis of 195 proteins differentially expressed in BHK cells infected with *Eimeria tenella*. Proteins were annotated by biological process, cellular component, and molecular function.

All DEPs were categorized using GO analysis based on the international standardized gene functional classification system. They were found to be involved in cellular (14.34%), metabolic (12.71%), single-organism processes (12.95%), and biological regulation (9.16%) (Fig. 2A). Additionally, some of these proteins were predicted to be membrane associated (9.49%), organelle associated (16.25%) and involved in macromolecular complexes (8.17%) (Fig. 2B). Moreover, proteins were assigned to structural molecule activity (6.76%), involved in binding (46.62%), catalysis (27.34%), transport (4.18%), molecular function regulator (6.76%), and transporter activity (5.07%) (Fig. 2C).

**Figure 3:**
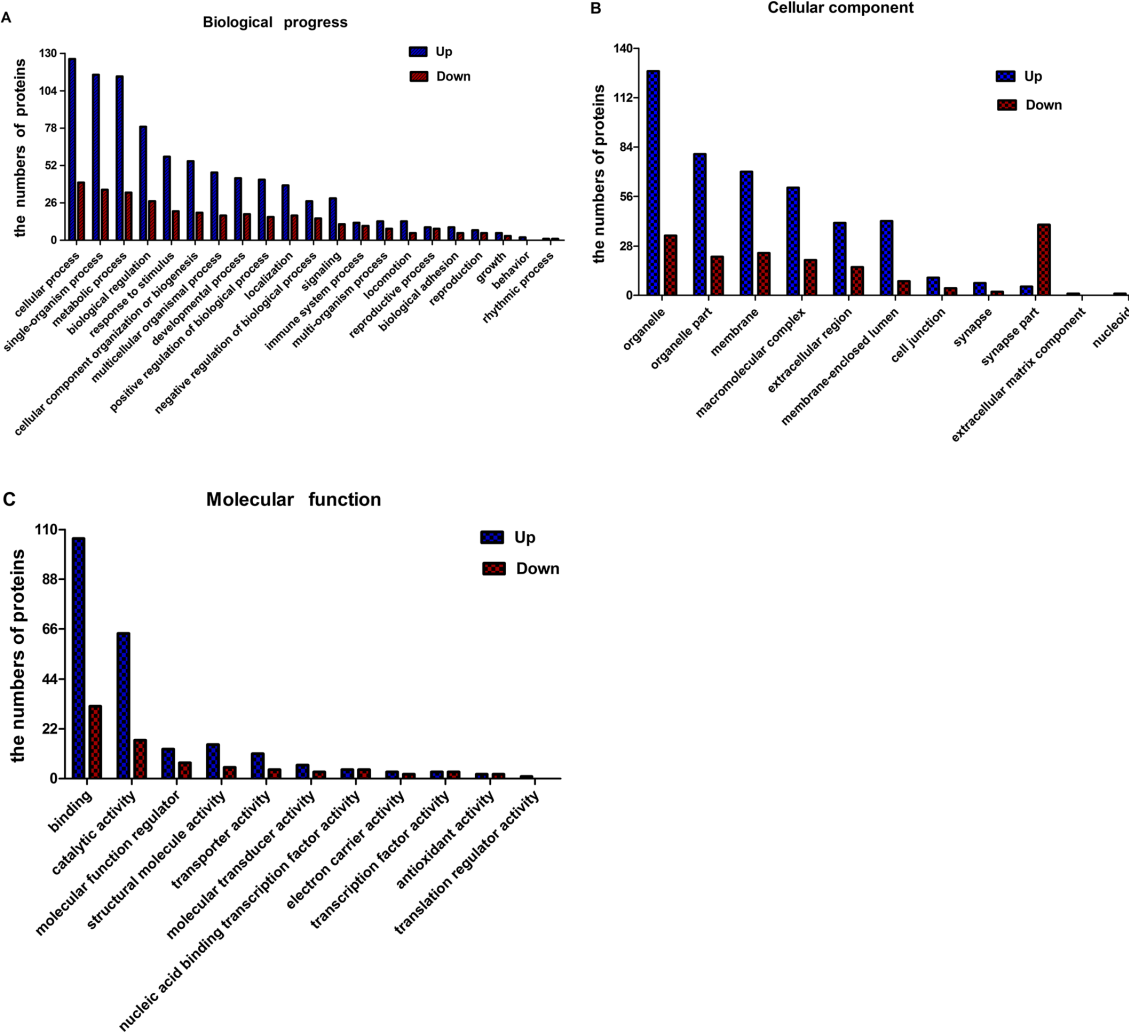
Gene ontology classification of up- and down- regulated proteins in BHK cells infected with *Eimeria tenella*. Blue bars indicate up-regulated proteins, red bars indicate down-regulated proteins.

Likewise, the significantly up- and down-regulated DEPs were also annotated with GO analysis. Interestingly, the proteins assigned to biological process and cellular component for the up-regulated proteins were similar to those assigned to the down-regulated DEPs. The three main biological process groups that the proteins identified were assigned to were cellular process, single-organism process, and metabolic process (Fig. 3A). Additionally, the cellular component groups assigned to both the up- and down-regulated proteins were membrane, organelle part and organelle (Fig. 3B). However, there are some differences in the molecular function between the up-regulated and down-regulated proteins. The up-regulated proteins were found to be mainly involved in binding and catalytic activity, while down-regulated proteins were found to be related to catalytic activity and molecular function regulators (Fig. 3C).

**Figure 4:**
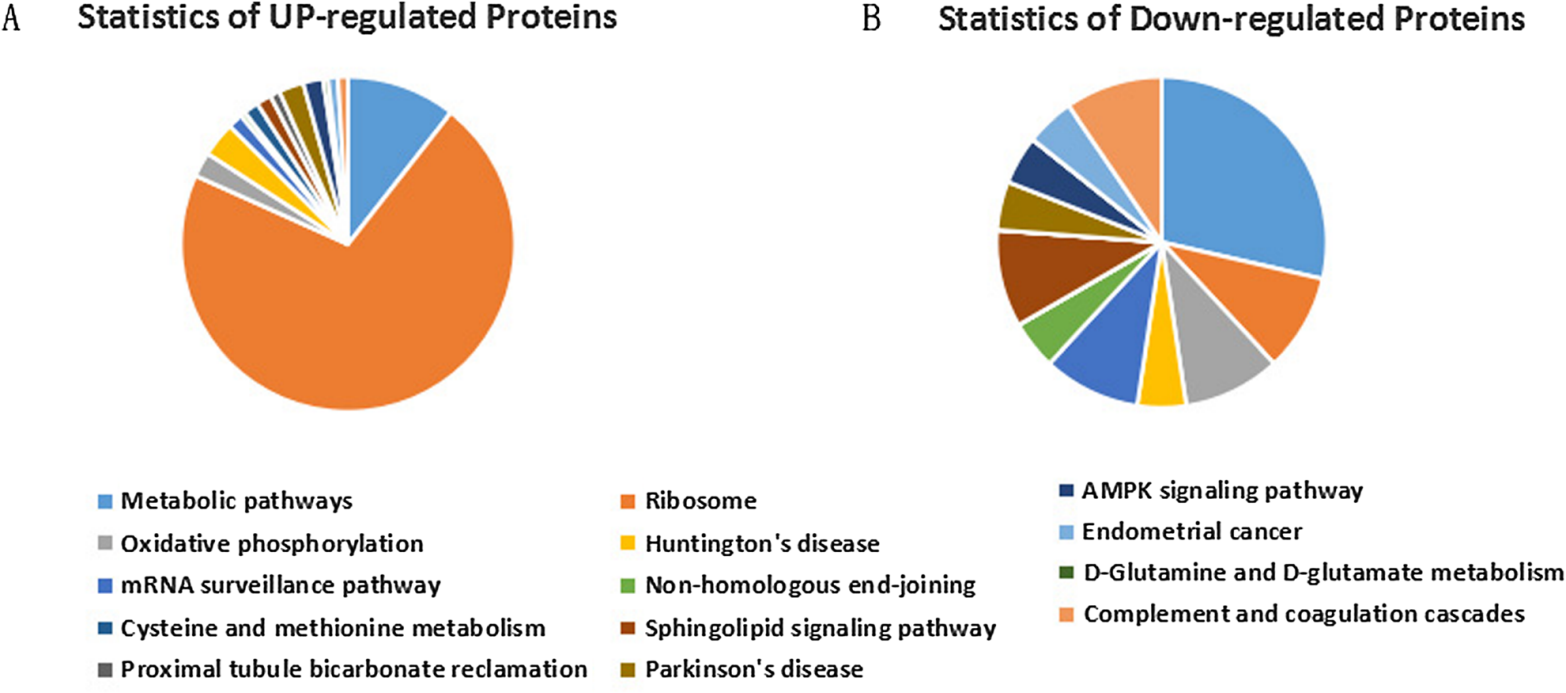
KEGG pathways classification of up- and down- regulated proteins in BHK cells infected with *Eimeria tenella*, including up-regulated proteins and down-regulated proteins.

The KEGG pathway analysis for the DEPs showed that they were involved in the PI3K-Akt, chemokine, RIG-I receptor-mediated phagocytosis, Ras, Wnt, and p53 signaling pathways. However, a significant difference in the KEGG pathways identified was found between the up-regulated and down-regulated proteins; most of the up-regulated DEPs were associated with the ribosome and the down-regulated DEPs were mainly associated with the mRNA surveillance pathway (Fig. 4).

### Validation of protein identiﬁcation and quantiﬁcation with qPCR and western blot

To confirm the reliability of the iTRAQ results, 12 proteins were selected for confirmation using qPCR including six up-regulated ones and six down-regulated ones and two proteins by immunoblotting analysis, including one up-regulated one and one down-regulated one.

**Figure 5:**
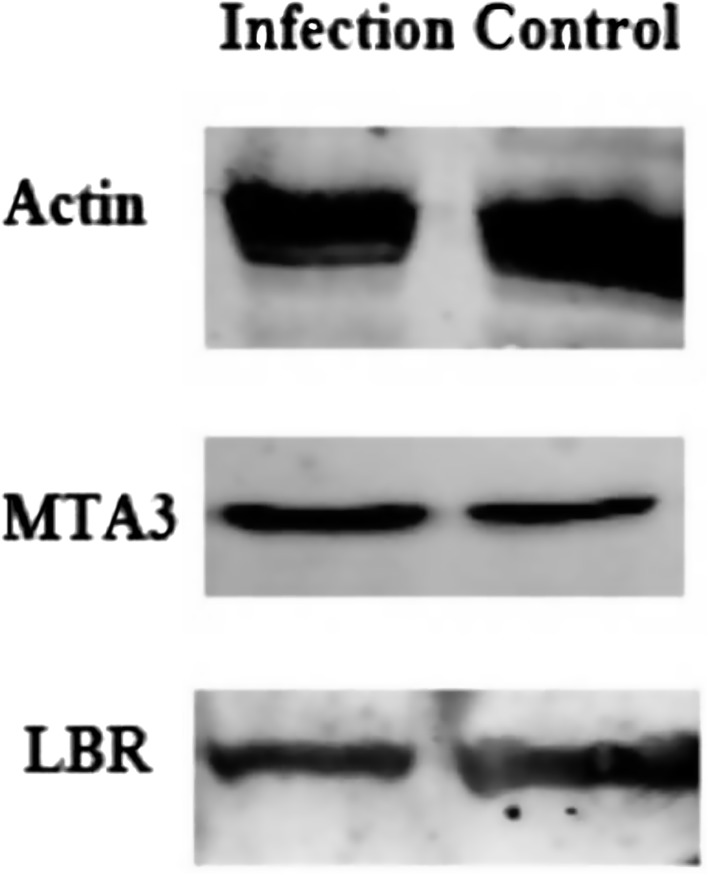
Western blot analysis of MAT3 and LBR in BHK cells infected with *Eimeria tenella* compared with mock-infected cells.

The mRNA expression levels detected with qPCR were consistent with those obtained by iTRAQ for 10 proteins including Atp51 (ATP synthase), Mapk9 (Mitogen-activated protein kinase 9), Pdss2 (Prenyl diphosphate synthase, subunit 2), Ppp2r4 (Protein phosphatase 2A), Gdi1 (Guanosine diphosphate dissociation inhibitor 1), Sec61a1 (Sec61 alpha 1 subunit), Tm7sf2 (Transmembrane 7 superfamily member 2), Rps8 (Ribosomal protein S8), Mrp120 (Mitochondrial ribosomal protein L20) and Tfrc (Transferrin receptor), with 83% agreement between the qRT-PCR and iTRAQ results. Results for two proteins did not agree with the iTRAQ data: Ptp4a1 (Protein phosphatase 4, regulatory subunit 1) and Bnip2 (BCL2/adenovirus E1B interacting protein 2) (Table 2). Therefore, taken together these results indicate that most proteins were regulated directly at the transcription level. However, some gene transcript levels did not match the levels of their corresponding proteins, which may indicate that the abundance of the protein may not depend only on the transcript level but also on post-translational modifications [19]. Additionally, one up-regulated protein (Metastasis associated gene 3, MTA3) and one down-regulated protein (Lamin B receptor, LBR) were selected to be analyzed with western blotting. These results confirmed the iTRAQ data, showing that MTA3 was clearly up-regulated in *E. tenella*-infected cells compared to mock-infected cells, while LBR was down-regulated (Fig. 5).

**Table 2:** Fold changes in the mRNA expression of 12 genes differently expressed in BHK cells infected with *Eimeria tenella*, detected by qPCR.

		Ratio				Ratio			
Accession	Gene name	iBR1	iBR2	iBR3	Average	qPCR1	qPCR2	qPCR3	Average
A0A061ILM3	Atp51	2.940	2.786	2.879	1.63	1.04	1.05	1.09	1.06
A0A1A6H7C7	Mapk9	1.382	1.388	1.374	1.38	1.98	2.13	2.13	2.08
G3HED1	Ptp4a1*	1.300	1.358	1.273	1.31*	0.24	0.36	0.30	0.30*
G3GRN2	Bnip2*	3.290	3.328	3.740	3.45*	0.60	0.68	0.82	0.70*
A0A061ILM3	Pdss2	2.940	2.786	2.879	2.87	1.38	1.49	1.69	1.52
G3II02	Ppp2r4	3.580	3.441	3.427	3.48	1.58	1.96	1.59	1.71
A0A061HU28	Gdi1	0.827	0.657	0.724	0.74	0.36	0.50	0.40	0.42
G3HIC4	Sec61a1	0.782	0.733	0.769	0.76	0.38	0.56	0.44	0.46
G3GRA0	Tm7sf2	0.796	0.705	0.697	0.73	0.08	0.06	0.10	0.08
A0A1A6GC37	Rps8	0.706	0.755	0.824	0.76	0.50	0.55	0.48	0.51
G3I429	Mrp120	0.771	0.797	0.731	0.76	0.58	0.66	0.80	0.68
A0A060BA20	Tfrc	0.722	0.830	0.740	0.76	0.56	0.74	0.80	0.70

* Expression patterns detected by qRT-PCR were not consistent with those obtained by iTRAQ.

## Discussion

Although *Eimeria* sporozoite motility and structural and secreted antigens appear to provide the mechanisms for propelling the sporozoite into the host cell, there is a growing body of evidence that the host cell provides characteristics by which the sporozoites recognize and interact with the host cell as a prelude to invasion [21]. After being infected with *Eimeria*, the host cells produce a corresponding change to deal with the damage with this infection. In this study, *E. tenella*-induced modulation of the host cell proteome at 24 hpi was analyzed by iTRAQ coupled with LC-MS/MS. The cell line BHK 21 used in this study has been widely used in *Eimeria* studies [28] and was better at supporting the growth of *E. tenella* than primary chicken or turkey cells [1]. The results of proteomic analysis showed that a total of 195 proteins were significantly changed in BHK 21 cells after being infected with *E. tenella* for 24 hpi.

A number of DEPs were predicted to be involved in the host signaling pathway. For instance, SOS1 was up-regulated in host cells infected with *E. tenella*, consistent with previous studies. Xiao et al. found that SOS1 had a significantly higher expression in human epithelial ovarian cancer than normal ovarian tissues and it played a role in promoting Ras activation in the Ras signaling pathway [34]. In contrast, some proteins are downregulated. Concerning menin and collagen alpha-1(XXVII) chain-like protein (Col24a1) Wu et al. found that reduced menin expression in human lung adenocarcinoma samples is associated with enhanced expression of Ras [33]. These data show that these proteins involved in the Ras signaling pathway play an important role in *Eimeria* invasion. KEGG analysis showed that the DEPs were also involved in PI3K-Akt, chemokine, and RIG-I receptor-mediated phagocytosis, and the Wnt and p53 signaling pathways. Hence, we predicted that *Eimeria* binding to the host surface doses not, by itself, guarantee access of the parasite into the intracellular environment and may require activation of specific signaling pathways critical for parasite entry into host cells.

It is well known that the intracellular stages of apicomplexan parasites, such as *Cryptosporidium parvum* [4, 9], *Theileria parva* [6, 10, 13], *Toxoplasma gondii* [2, 3, 15], *Neospora caninum* [8, 17, 18] and *Eimeria* [5], modulate host cell apoptosis to guarantee successful intracellular development. To date, only a few details are known about the molecular mechanisms allowing for long-term survival of *E. tenella* within adequate host cells. In this study, a total of 148 DEPs were found to be related to apoptotic pathways. The Frizzled-3 protein (Fzd3) is a known transmembrane receptor involved in the secretion of Wnt glycoproteins, important for Wnt signal transduction cascades. Khan et al. found that the activation of the Wnt/β-catenin pathway led to altered expression of genes involved in cell cycle regulation and apoptosis in normal and leukemic B-cell progenitors [12]. Additionally, Apoptosis-stimulating of p53 protein 2 (TP53BP2) could specifically regulate p53-dependent apoptosis and could be down-regulated with the micro RNA miR-548d-3p by directly targeting the 3′ UTR of the protein, showing that the miR-548d-3p/TP53BP2 pathway is critically involved in the proliferation and apoptosis of breast cancer cells [26]. Concerning our results, Fzd3 expression was decreased and TP53BP2 was increased in *E. tenella*-infected cells, and we therefore propose that they could trigger cell apoptosis to defend against *Eimeria* invasion.

The parasite’s successful invasion of a host cell depends entirely on the interaction between the parasite and the host cell membrane. It has been shown that the specific glycoprotein receptor on the erythrocyte surface could recognize *Plasmodium falciparum* invading cells [16]. In this study, 178 membrane proteins were found to be altered in BHK-21 cells infected with *E. tenella*. These included amino acid transporters (SLC1A1), mitochondrial membrane ATP synthase (ATP5l) and cytochrome c oxidase polypeptide 7A1 (COX7A1). It has been found that IL-2 can up-regulate the expression of amino acid transporters such as SLC1A1, which then stimulates natural killer cells to defend against pathogen invasion [11]. Therefore, the identification of these important membrane-associated proteins in this work indicates that membrane proteins contributed to resistance against *E. tenella* infection.

Some DEPs were also predicted to be involved in the host metabolic and stress and defense response, such as apoptosis antagonizing transcription factor (AATF), cyl-CoA, lysophosphatidylglycerol acyltransferase 1 (Lpgat1), platelet-activating factor acetylhydrolase IB subunit beta (Pafah1b2), and acid ceramidase (Asah1). These data indicate that host metabolism was strongly affected after the cells were infected with *E. tenella* and the cells produced many antimicrobial peptides as part of the innate immune response to defend against *E. tenella* infection.

## Conclusion

To sum up, this is the first attempt to study the proteomic changes in *E. tenella*-infected BHK-21 cells through an iTRAQ-based proteomic method. Our analyses of the DEPs were comprehensive, but further functional studies are needed to clarify the pathogenic mechanisms and cellular responses to *E. tenella* infection. This could be important to understand the interaction between the *Eimeria* parasite and its host cell.

## Supplementary material (Table S1)

Details list of proteins of BHK cells infected with *Eimeria tenella*.
Details list of 195 proteins differentially expressed in BHK cells infected with *Eimeria tenella*. Supplementary materials are available at https://www.parasite-journal.org/10.1051/parasite/2019009/olm.

## Competing interests

The authors declare that they have no competing financial interests.
